# Cell-Free DNA for Genomic Analysis in Primary Mediastinal Large B-Cell Lymphoma

**DOI:** 10.3390/diagnostics12071575

**Published:** 2022-06-28

**Authors:** Alfredo Rivas-Delgado, Ferran Nadeu, Marcio Andrade-Campos, Cristina López, Anna Enjuanes, Pablo Mozas, Gerard Frigola, Luis Colomo, Blanca Sanchez-Gonzalez, Neus Villamor, Sílvia Beà, Elías Campo, Antonio Salar, Eva Giné, Armando López-Guillermo, Beatriz Bellosillo

**Affiliations:** 1 Hematology Department, Hospital Clínic de Barcelona, 08036 Barcelona, Spain; mozas@clinic.cat (P.M.); egine@clinic.cat (E.G.); alopezg@clinic.cat (A.L.-G.); 2 Institut d’Investigacions Biomèdiques August Pi i Sunyer (IDIBAPS), 08036 Barcelona, Spain; nadeu@clinic.cat (F.N.); clopez2@clinic.cat (C.L.); anna.enjuanes@idibaps.org (A.E.); villamor@clinic.cat (N.V.); sbea@clinic.cat (S.B.); ecampo@clinic.cat (E.C.); 3 Faculty of Medicine and Health Sciences, Universitat de Barcelona, 08007 Barcelona, Spain; 4 Centro de Investigación Biomédica en Red de Cáncer (CIBERONC), 28029 Madrid, Spain; 5 Hematology Department, Hospital del Mar-IMIM, 08003 Barcelona, Spain; 64138@parcdesalutmar.cat (M.A.-C.); bsanchezgonzalez@psmar.cat (B.S.-G.); asalar@psmar.cat (A.S.); 6 Grup de Recerca Clínica, Aplicada en Neoplàsies Hematològiques-Hospital del Mar-IMIM, 08003 Barcelona, Spain; bbellosillo@psmar.cat; 7 Hematopathology Section, Pathology Department, Hospital Clínic de Barcelona, 08036 Barcelona, Spain; frigola@clinic.cat; 8 Pathology Department, Hospital del Mar-IMIM, 08003 Barcelona, Spain; lcolomo@psmar.cat; 9 Department of Medicine and Life Sciences, Universitat Pompeu Fabra, 08002 Barcelona, Spain

**Keywords:** cell-free DNA, primary mediastinal large B-cell lymphoma, mutational profile, copy number analysis

## Abstract

High-throughput sequencing of cell-free DNA (cfDNA) has emerged as a promising noninvasive approach in lymphomas, being particularly useful when a biopsy specimen is not available for molecular analysis, as it frequently occurs in primary mediastinal large B-cell lymphoma (PMBL). We used cfDNA for genomic characterization in 20 PMBL patients by means of a custom NGS panel for gene mutations and low-pass whole-genome sequencing (WGS) for copy number analysis (CNA) in a real-life setting. Appropriate cfDNA to perform the analyses was obtained in 18/20 cases. The sensitivity of cfDNA to detect the mutations present in paired FFPE samples was 69% (95% CI: 60–78%). The mutational landscape found in cfDNA samples was highly consistent with that of the tissue, with the most frequently mutated genes being *B2M* (61%), *SOCS1* (61%), *GNA13* (44%), *STAT6* (44%), *NFKBIA* (39%), *ITPKB* (33%), and *NFKBIE* (33%). Overall, we observed a 75% concordance to detect CNA gains/losses between DNA microarray and low-pass WGS. The sensitivity of low-pass WGS was remarkably higher for clonal CNA (18/20, 90%) compared to subclonal alterations identified by DNA microarray. No significant associations between cfDNA amount and tumor burden or outcome were found. cfDNA is an excellent alternative source for the accurate genetic characterization of PMBL cases.

## 1. Introduction

Primary mediastinal large B-cell lymphoma (PMBL) is recognized as a specific entity by the World Health Organization (WHO) classification, with particular clinical, histological, and molecular features. It accounts for 2% to 3% of all non-Hodgkin lymphomas [1]. Typically, patients present with a large mass in the anterior mediastinum, which often makes it difficult to perform a biopsy. Occasionally, histological samples can initially be non-diagnostic due to extensive fibrosis and necrosis, leading to patients either undergoing mediastinoscopy or thoracoscopy to reach the diagnosis [2]. PMBL has recurrent genomic alterations, including somatic gene mutations and copy number alterations (CNA), as well as a characteristic gene expression profile. Constitutive activation of the nuclear factor-kB (NF-kB) and JAK/STAT pathways are recognized as a hallmark of this disease [3,4].

Cell-free DNA (cfDNA) has emerged as a noninvasive tool, complementary to tissue biopsies, particularly in cases in which a tumor biopsy is clinically difficult to obtain [5]. In Oncology, cfDNA has demonstrated its utility in monitoring the response to treatment real time, guiding therapy, and detecting early recurrence [6]. In recent years, cfDNA has been investigated in Hodgkin and non-Hodgkin lymphomas, using next-generation sequencing (NGS) for genetic analysis, providing a straightforward and easier detection method for assisting in the molecular profiling of tumors. In addition, cfDNA baseline levels have proven to be a remarkably useful tool to predict response to treatment and clinical outcomes [7]. Several studies of cfDNA in diffuse large B-cell lymphoma (DLBCL) using NGS customized gene panels have shown that the mutational landscape from cfDNA samples was highly consistent with that observed in tissue biopsies, and also highlighted mutations only present in cfDNA, a fact that could be explained by the spatial heterogeneity of the tumor [8,9,10,11]. Although some of these studies include PMBL [9,11,12], the low number of cases analyzed and the lack of PMBL-specific analyses preclude any solid conclusion about the use of cfDNA for PMBL genomic characterization.

Genetic studies are difficult in PMBL mainly due to the scarce available material. This is particularly relevant both at diagnosis, when the biopsy is obtained by a large core needle leading to sufficient material for establishing a diagnosis but not for further analysis, and at relapse. In this setting, the use of cfDNA might overcome this limitation and become a reliable ground for genetic studies in PMBL. Thus, the aim of this study was to assess the use of cfDNA as a reliable source for genomic characterization using a custom NGS panel for gene mutations and low-pass whole-genome sequencing (WGS) for CNA in newly diagnosed patients with PMBL in a real-life setting and its correlation with clinical parameters.

## 2. Methods

### 2.1. Patients

Twenty-four patients were diagnosed with PMBL in two institutions between 2015 and 2020. Using the availability of plasma for cfDNA assessment, we selected the 20 cases with available plasma samples at diagnosis. The mutational profile could be evaluated in cfDNA in 18 cases with enough cfDNA quantity after extraction (at least 15 ng of cfDNA for library construction), which constituted the subjects of the present study. 

Staging was performed according to standard procedures, including positron emission tomography/computed tomography (PET/CT) and unilateral bone marrow biopsy [13]. The main clinico-biological and follow-up characteristics were recorded and analyzed (variables studied are detailed in Supplementary Methods) (Table 1). All patients were treated with chemoimmunotherapy, including R-CHOP (rituximab, cyclophosphamide, doxorubicin, vincristine, and prednisone) or DA-EPOCH-R (dose-adjusted etoposide, prednisone, vincristine, cyclophosphamide, doxorubicin, and rituximab), followed by consolidative radiation therapy in selected cases (Table 1). Responses were assessed by end-of-therapy PET/CT according to standard guidelines [13].

### 2.2. Histologic Review

Cases were reviewed for the present study by G.F., L.C., and E.C. Morphological and immunohistochemical analyses were carried out according to the WHO classification [1] using the following markers: (1) B-cell differentiation antigens: CD10, CD19, CD20, CD22, CD23, CD79a, BCL6, and IRF4/MUM1, (2) T-cell antigens: CD2, CD3, CD4, CD5, CD7, CD8, and CD45RO, and (3) cell proliferation and apoptosis: Ki67, CD30, and BCL2.

### 2.3. Sample Collection and DNA Extraction

Plasma samples were collected at diagnosis, before the start of treatment, using EDTA tubes or PAXgene Blood ccfDNA tubes (PreAnalytiX, Hombrechtikon, Switzerland) and were processed within the first four hours after blood extraction (Supplementary Methods). cfDNA was extracted from 2–4 mL of plasma using the QIAamp circulating nucleic acid kit (Qiagen, Hilden, Germany) or the MagMax Cell Free DNA isolation kit (Thermo Fisher Scientific, Waltham, MA, USA). In parallel, genomic DNA (gDNA) was isolated from diagnostic formalin-fixed paraffin-embedded (FFPE) tissue biopsies using the AllPrep DNA/RNA FFPE Kit (Qiagen, Hilden, Germany) according to the manufacturer’s instructions.

Levels of cfDNA are reported as haploid genome equivalents per mL of plasma (hGE/mL), determined as the product of total cell-free DNA concentration and the mean allele fraction of somatic alterations, and expressed as a base-10 logarithm (log hGE/mL). We selected the 2.5 log hGE/mL threshold for pretreatment cfDNA level according to previous publication [12].

### 2.4. Mutational Profile and Copy Number Alterations

NGS was performed using a panel of 112 recurrently mutated genes in B-cell lymphoma as previously described (Supplementary Table S1) [10]. Briefly, libraries were performed with 15–30 ng of cfDNA and 150 ng of gDNA using molecular-barcoded library adapters (ThruPLEX Tag-seq kit; Takara, Tokyo, Japan) coupled with a custom hybridization capture-based method (SureSelect XT Target Enrichment System Capture strategy, Agilent Technologies Inc., Santa Clara, CA, USA) and sequenced in a MiSeq instrument (Illumina, San Diego, CA, USA, 2 × 150 bp) (Supplementary Methods). The bioinformatic analysis was performed using our in-house NGS pipeline [10,14,15]. Synonymous and intronic variants, as well as potential polymorphisms, were removed from downstream analyses [10]. A detailed description on sample processing, library preparation, sequencing, and bioinformatic analysis can be found in the Supplementary Files.

Libraries for low-pass WGS were performed with the ThruPLEX Tag_seq kit using 30 ng of cfDNA in only two cases (cases 4 and 5) due to the availability of cfDNA for library constructions. Libraries were sequenced in a NextSeq550 (2 × 75 bp, Illumina) aiming at mean coverage of 0.5×. Raw reads were mapped to the human reference genome (GRCh37) using the BWA-mem algorithm (v0.7.17) [16]. BAM files were generated, sorted, and indexed using samtools (v1.9) [17]. PCR duplicates were flagged using Picard tools (v2.17.0). FastQC (v0.11.5) and Picard tools were used to extract quality control metrics. Mean coverage was 0.97× [range 0.34–2.41×]. CNA were extracted using ichorCNA (v0.3.2) [18] following authors’ recommendations. Copy number neutral -loss of heterozygosity (CN-LOH) were not evaluated using low-pass WGS.

CNA were also analyzed from gDNA using the Affymetrix Genome-wide Oncoscan CNV FFPE arrays. Gains, losses and copy neutral loss of heterozygosity (CN-LOH) were evaluated using Nexus version 9.0 Discovery Edition software (Biodiscovery, El Segundo, CA, USA) using the GRCh37 human reference genome. CNA with a minimum size of 100 kb and telomeric CN-LOH larger than 10 Mb were considered.

### 2.5. Statistical Analyses

We used standard definitions for complete response (CR), progression-free survival (PFS), and overall survival (OS) [13]. The chi-square method was used to compare categorical variables and the Student’s *t*-test for continuous variables. Non-parametric tests were applied when necessary. Logistic regression was used to select the best variables predicting for CR. Actuarial survival analysis was performed by the Kaplan–Meier method and differences were assessed by the log-rank test. Statistical analyses were carried out using R (version 3.6.3; R Foundation, Vienna, Austria).

## 3. Results

### 3.1. Detection of Genetic Alterations in cfDNA

**Figure 1:**
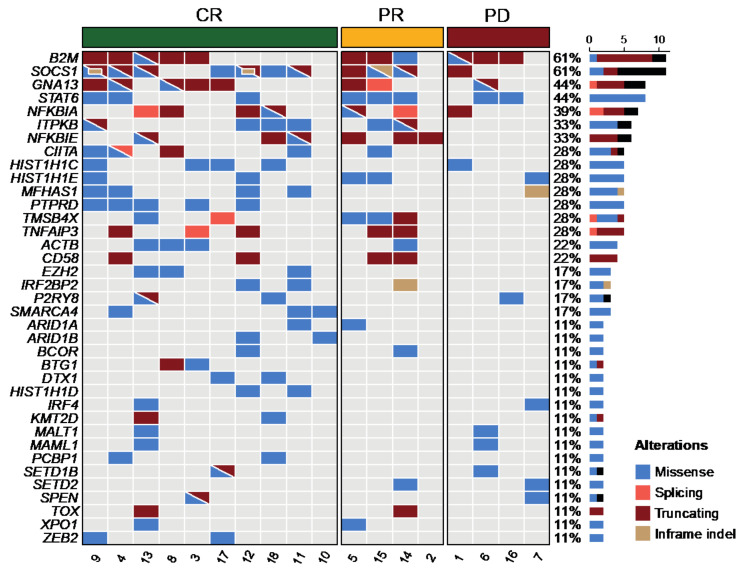
Mutational profile in the cfDNA of the 18 patients with PMBL. Each column represents one tumor sample, and each row represents one gene. Cases are grouped by response to first-line treatment.

cfDNA was obtained in 20 patients. However, as previously indicated, the mutational profile of two cases could not be assessed due to the low amount of cfDNA or low quality of DNA (one case each). Thus, the mutational landscape was finally assessed in 18 cases with a mean coverage of the cfDNA samples of 363× (range: 114–616×). The median number of mutations per sample was 15 (range: 1–30). Figure 1 shows the mutational profile of all the patients, restricted to genes mutated in ≥2 cases (more than 10%). The most frequently mutated genes were *B2M* (61%), *SOCS1* (61%), *GNA13* (44%), *STAT6* (44%), *NFKBIA* (39%), *ITPKB* (33%), and *NFKBIE* (33%). The complete list of mutations is detailed in Supplementary Table S2.

### 3.2. Validation of Mutations in Tissue Biopsies

We performed targeted NGS in paired FFPE samples to validate the cfDNA mutational analysis. Sufficient DNA for library constructions could be obtained from FFPE samples in 9 out of 18 cases. The remaining 9 cases were excluded due to insufficient material for DNA extraction (*n* = 6) or insufficient quantity and/or quality for library preparation (*n* = 3). 

**Figure 2:**
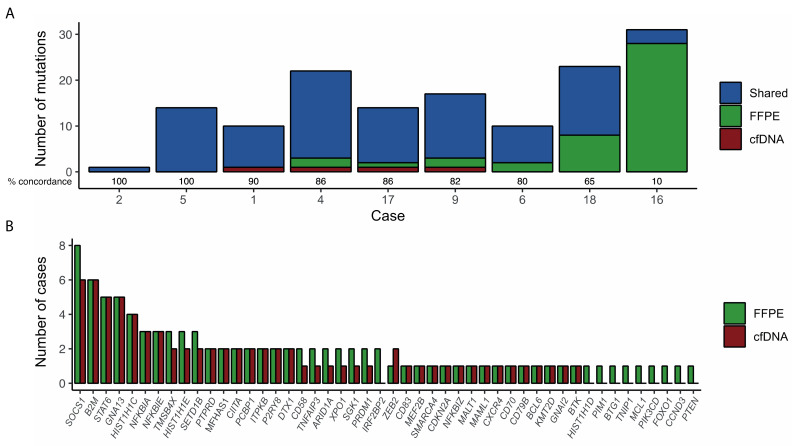
Concordance between mutations detected in cfDNA and matched tumor gDNA. (**A**) Number of mutations by case. Mutations are coded by color according to whether they were detected in both samples (blue), only in the FFPE sample (green), or only in cfDNA (red). The percentage of concordance is showed for each case; (**B**) Prevalence of somatic mutations detected by NGS in cfDNA and gDNA.

The mean coverage in FFPE samples was 687× (range: 110–1520×). In 7 out of 9 cases (78%), most mutations (>80%) were observed both in the cfDNA and the FFPE samples. In the remaining 2 cases, the number of mutations identified in cfDNA was lower than that observed in the paired FFPE sample. Of note, these 2 cases corresponded to localized disease (Ann Arbor stage I). In 4 cases, additional mutations were only detected in cfDNA compared to gDNA (Figure 2). Overall, the sensitivity of cfDNA to detect the mutations present in paired FFPE samples was 69% (95% CI: 60–78%).

### 3.3. Copy Number Alterations

**Figure 3:**
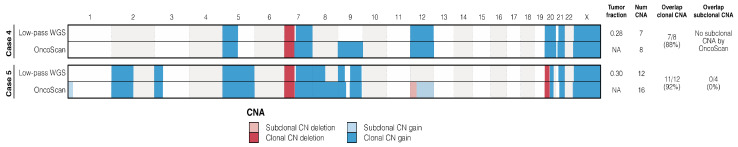
Copy number profile of primary mediastinal large B-cell lymphoma. The first line refers to CNA from cell-free DNA low-pass WGS, followed by the CNA of FFPE Oncoscan. Chromosomes are sorted from 1 to X and p to q (chromosome Y was excluded). Gains and losses are depicted in blue and red, respectively. The subclonal CNA are highlighted by low color brightness.

We investigated CNA using OncoScan arrays in 2 FFPE samples. Several CNA were detected in both samples, corresponding to a total of 8 and 16 alterations per case (Supplementary Table S3). In both cases, we observed gains of 5p, 7q, and trisomy 9, 21, and X, as well as losses of 7p. We compared the CNA results from the arrays with the CNA obtained from the low-pass WGS of cfDNA. Overall, we observed a 75% concordance to detect CNA between OncoScan and low-pass WGS. The sensitivity of low-pass WGS was remarkably higher for clonal CNA (18/20, 90%) compared to subclonal alterations identified by OncoScan (absolute probe median ≤0.1; 0/4 alterations) (Figure 3).

### 3.4. Tumor Burden Assessment by cfDNA

The median amount of cfDNA was 2.65 log hGE/mL (range, 1.77–3.60). There was no significant association between the amount of cfDNA and the presence of B symptoms, elevated LDH, advanced Ann Arbor stage, or the presence of a bulky mass. Of note, the number of detected mutations did not correlate with the amount of cfDNA (mean 7.2 vs. 7.8 for low and high cfDNA, respectively).

### 3.5. Clinical Features, Treatment, and Outcome of the Patients

The main clinico-biological features of the 18 patients in whom mutational analyses were performed are listed in Table 1. Seventy-eight percent of patients had bulky disease, 33% had advanced stage (III/IV), and the majority (84%) showed low- or low-intermediate-risk International Prognostic Index. After frontline treatment, 10 (56%) patients achieved a CR, 4 (22%) partial response and 4 (22%) were refractory. None of the patients achieving a CR relapsed during follow-up. None of the initial clinical variables predicted for the obtention of a CR, nor did the total number of gene mutations. *PTPRD* mutation predicted for a higher CR rate (100% mutated vs. 39% wild-type; *p* = 0.029). After a median follow-up of 44 months, 3-year PFS was 56% (95% CI: 37–84%). No clinical variable predicted PFS, whereas the single mutation at *PTPRD* predicted for a longer PFS (2-year PFS 100% vs. 39% for mutated and wild-type *PTPRD*, respectively; *p* = 0.035). Overall, three patients died during follow-up, with a 3-year OS of 85% (95% CI: 63–99%). No clinical or genetic variables were able to predict OS. 

## 4. Discussion

Analysis of cfDNA has been increasingly used for assessing molecular profiling at diagnosis, to define prognosis, and for identification of therapeutic targets in oncology, including patients with lymphoma [19]. Here, we have analyzed the applicability of cfDNA as a reliable source for mutational and CNA assessment of PMBL patients in whom diagnostic biopsies are often difficult to obtain. Indeed, the information about the utility of cfDNA in PMBL is scarce, with only one recently published report [20].

In the present series, we were able to detect mutations in the cfDNA in 18 out of 20 (90%) cases, a similar rate to that reported in other lymphoma series, including a DLBCL cohort from our own institution [10,11]. Camus et al. [20] reported the mutational profile of 44 patients with PMBL using an abridged targeted panel of nine genes, detecting at least one mutation in 32 (73%) patients. The higher detection rate obtained in our study could be explained by the fact that we have used a broader gene panel including analysis of CNA and we have used molecular-barcode technology to improve background removal in the analysis of sequencing data and increase sensitivity. We also aimed to determine the reliability of the technique by comparing the mutational profile obtained from FFPE samples. In our hands, the sensitivity of cfDNA to detect mutations present in the paired FFPE samples was 69%. Once more, this is in line with previous publications on DLBCL by us and others [9,10,11].

Different genetic alterations have been described in PMBL, including constitutive activation of the NF-ĸB and JAK-STAT pathways, along with genetic alterations that promote immune evasion [21]. The mutational landscape described from cfDNA in our study was highly consistent with that previously published in different series, including two integrative genetic analyses [3,22]. Thus, members of the JAK-STAT and NF-kB pathways, including *SOCS1*, *STAT6*, *NFKBIA*, *NFKBIE*, and *TNFAIP3* were among the most frequently altered genes. Recurrent CNA have been described in PMBL including gains of 9p (*CD274* and *PDCD1LG2*), 2p (*REL*), chromosome 6, and 11q. Importantly, we were also able to detect CNA from cfDNA using low-pass WGS, with high accuracy for clonal CNA compared to matched FFPE tumoral tissue samples. The usefulness of cfDNA for CNA has been previously assessed on lymphoma. Rushton and colleagues [23] evaluated CNA from 45 relapsed/refractory DLBCL (rrDLBCL) derived liquid biopsies collected after relapse using low-pass WGS, identifying nine regions enriched for recurrent amplifications or deletions among rrDLBCL, providing insight into the biology of rrDLBCL from easily accessible sources such as the peripheral blood.

Due to the limited number of fully studied cases, it was difficult to find significant correlations with prognosis in the current study. In fact, even the main clinical variables did not show a predictive impact. Nevertheless, it is noteworthy that patients with *PTPRD* mutation had a higher CR rate and prolonged PFS. *PTPRD* encodes the receptor-type-protein-tyrosine-phosphatase-δ, a tumor suppressor gene involved in cell growth regulation through the JAK-STAT signal pathway. It has been previously described in indolent and aggressive lymphomas, including marginal zone lymphoma and the primary central nervous system DLBCL [24]. *PTPRD* mutations were associated with better PFS and OS in patients with non-small cell lung cancer treated with immune checkpoint blockade, providing evidence for exploring the role of this mutation in the PMBL patients who receive immune checkpoint blockade in the era of immunotherapy [25]. 

Besides the genetic characterization of the mutational profile and CNA, the quantification of cfDNA has been associated with well-recognized clinical parameters of tumor burden (LDH, beta-2-microglobulin, total metabolic tumor volume), CR rate, and survival in DLBCL and other lymphoid malignances, including a series from our institution [9,10,12]. However, in the present series, this correlation could not be established.

Tissue biopsies represent a bottleneck in the genetic characterization of the PMBL due to the location of the tumors. Surgical biopsy by cervical mediastinoscopy, anterior mediastinotomy, or thoracoscopy is preferred over core biopsy [26], and sometimes samples are scarce or exhausted during the diagnostic process, limiting further molecular studies [27]. Plasma is an accessible source of tumor DNA when DNA cannot be retrieved from the diagnostic biopsy tissue and, indeed, cfDNA might be useful not only at diagnosis (when tissue biopsy is mandatory), but also upon relapse, when excision biopsy is frequently unavailable. In this sense, our study supports the use of cfDNA for an accurate genetic characterization of PMBL tumors. 

## Figures and Tables

**Table 1:** Main baseline features, treatment, and response of the 18 patients with PMBL.

Characteristics	N (%)
Median age (range)	30 (19–68)
Female/Male	11/7 (61/39)
ECOG-PS ≥ 2	2 (11)
B symptoms	8 (44)
Stage	
I/II	12 (67)
III/IV	6 (33)
Bone marrow infiltration	0 (0)
Bulky mass (>7 cm)	14 (78)
Lactate dehydrogenase > normal	14 (78)
IPI	
Low risk	10 (56)
Low-Intermediate risk	5 (28)
High-Intermediate risk	2 (11)
High risk	1 (5)
Treatment	
R-CHOP	13 (72)
DA-R-EPOCH	5 (28)
Consolidative radiotherapy	10 (56)
Response to treatment	
Complete response	10 (56)
Partial response	4 (22)
Progressive disease	4 (22)

ECOG: Eastern Cooperative Oncology Group. IPI: International Prognostic Index. R-CHOP: rituximab, cyclophosphamide, doxorubicin, vincristine, and prednisone. DA-EPOCH-R: dose-adjusted etoposide, prednisone, cyclophosphamide, vincristine, doxorubicin, and rituximab.

## Data Availability

The datasets analyzed during the current study are available from the corresponding author on reasonable request.

## Supplementary Materials

The following supporting information can be downloaded at: https://www.mdpi.com/article/10.3390/diagnostics12071575/s1. Supplementary methods; Table S1. Genes sequenced using target NGS; Table S2. All mutations (374) called in series of 18 PMBCL after exclusion of synonymous variants, intron variants and known polymorphisms. Table S3. CNA in PMBCL.
