# Supplementary material for: Cell-Free DNA for Genomic Analysis in Primary Mediastinal Large B-Cell Lymphoma
Source: Diagnostics (Basel). 2022 Jun 28;12(7):1575. doi: 10.3390/diagnostics12071575 (PMC9324191; doi:10.3390/diagnostics12071575)
Supplement: Supplementary file 1 [file diagnostics-12-01575-s001.zip › Supp Files/diagnostics-1749759 Supp Methods R1.pdf]

Cell-Free DNA for Genomic Analysis in Primary Mediastinal Large B-Cell Lymphoma

Supplemental Materials

Supplementary Methods

1. Clinical and evolutionary characteristics
2. Sample processing and DNA extraction
3. Library design for hybrid selection
4. Next generation sequencing
5. Bioinformatic analyses
6. Somatic *versus* germ line variants classification

Clinical and evolutionary characteristics

The main clinico-biological and evolutionary characteristics were recorded and analyzed. These variables included: (a) clinical data: age, sex, performance status according to the Eastern Cooperative Oncology Group (ECOG) scale, presence of B symptoms and bulky disease (defined as a tumor diameter >7 cm), nodal and extranodal involvement, number of extranodal involved sites, palpable splenomegaly, bone marrow infiltration, and Ann Arbor Stage; (b) hematological and biochemical parameters: white blood cell and lymphocyte counts, hemoglobin, and serum lactate dehydrogenase (LDH); (c) the International Prognostic Index (IPI).

Sample processing and DNA extraction

Blood samples were centrifuged for 10 min at 1500g and subsequently for 1 minute at 20000 g. Plasma was aliquoted into 1mL in microtubes and stored at −80°C until extraction. cfDNA was extracted from 1-4 mL of plasma using the QIAamp circulating nucleic acid kit (Qiagen, Germany) or MagMAX Cell-Free DNA Isolation Kit (ThermoFisher Scientific, Foster City, USA). The quantity and quality of the samples were assessed using Qubit High Sensitivity dsDNA (Thermo Fisher Scientific) and TapeStation (Agilent). Genomic DNA and RNA were isolated from FFPE diagnostic tissue biopsies. Five 10 μm-thick sections per sample were used to extract RNA and DNA using the AllPrep DNA/RNA FFPE Kit (Qiagen, Germany) according to the manufacturer's instructions.

Library design for hybrid selection

A capture-based sequencing gene panel targeting112 genes (target region: 388Kb) recurrently mutated in DLBCL and other mature B-cell tumors was specifically designed for this project (Supplementary Table S1). The design of the panel was carried out to interrogate: 1) genes mutated in >5% of mature B-cell tumors, 2) genes associated with resistance to chemotherapy in mature B-cell tumors.

Next generation sequencing

Mutation profiles were generated using a custom hybridization capture-based panel strategy (compatible with cfDNA and DNA from FFPE samples) and subsequent sequenced in a MiSeq instrument (Illumina). Libraries were performed using 15-30 ng of cfDNA and 150 ng of gDNA from FFPE samples, following the procedure indicated by the manufacturer recommendations. Targeted sequencing was performed using

molecular-barcoded library adapters (*ThruPLEX Tag-seq* kit, Takara) and a hybridization capture based method (SureSelectXT-Agilent Technologies), following the manufacturer's recommendations. Quality of the libraries was determined using the Bioanalyzer high sensitivity DNA kit (Agilent) and quantified by PCR using the KAPA library quantification kit (KAPA Biosystems). Finally, the libraries were pooled and sequenced at 2x150 bp in a MiSeq instrument.

Bioinformatic analyses

Capture-based next-generation sequencing data was analyzed for gene mutations using an updated version of our in-house pipeline[1]. Briefly, raw reads were trimmed using the SurecallTrimmer (v4.0.1, AGeNT, Agilent). Alignment of the trimmed reads was performed using BWA-mem algorithm (v0.7.17), PCR or optical duplicates were marked using MarkDuplicates from Picard (RRID: SCR_006525), and the base quality score recalibration was performed using GATK's BaseRecalibrator and ApplyBQSR functions (RRID: SCR_001876 v4.0). Coverage uniformity and quality control metrics were extracted using Picard (CollectInsertSizeMetrics, EstiamteLibraryComplexity, CollectAlignmentSummaryMetrics, CollectTargetedPcrMetrics, and CollectSequencingArtifactMetrics) and samtools (idxstats, and flagstat). As previously observed[2], all interrogated regions showed sufficient sequencing quality and coverage. Variant calling was performed in parallel using VarScan2 (v2.4.3), Mutect2 (GATK v.4.0.4.0), VarDict (v1.4), outLyzer (v1.0), and freebayes (v.1.1.0-54-g49413aa). Only variants that were identified as "PASS" by at least 3 of the algorithms were considered. Finally, variants were annotated using snpEff/snpSift (v4.3t). Mutations were visually

inspected on Integrative Genomics Viewer (IGV). All programs were executed using default settings.

Somatic *versus* germ line variants classification

Variants reported in 1000 Genome Project, ExAC and/or gnomAD with a population frequency >1% were considered polymorphisms and automatically removed from the analysis. To further filter out non-recurrent polymorphisms, variants were only considered somatic if 1) they were not reported as germ line in our custom ICGC data base of 506 WGS/WES[3]; and were 2) truncating, or 3) predicted as potentially damaging by at least one of the following algorithms: CADD (phred score > 10), PolyPhen2 (score> 0.9), SIFT (score < 0.1) (RRID: SCR_012813), and/or MutationAssessor (score > 2) (RRID: SCR_005762).

REFERENECS

1. Nadeu F, Delgado J, Royo C, et al. Clinical impact of clonal and subclonal TP53, SF3B1, BIRC3, NOTCH1, and ATM mutations in chronic lymphocytic leukemia. *Blood*. 2016; 127(17)**:** 2122–2130.
2. Rivas-Delgado A, Nadeu F, Enjuanes A, et al. Mutational Landscape and Tumor Burden Assessed by Cell-free DNA in Diffuse Large B-Cell Lymphoma in a Population-Based Study. *Clinical Cancer Research.* 2021 27(2): 513–521.
3. Puente XS, Beà S, Valdés-Mas R, et al. Non-coding recurrent mutations in chronic lymphocytic leukaemia. *Nature.* 526, 519–524 (2015).
